# Untargeted and Targeted Metabolomic Profiling of Australian Indigenous Fruits

**DOI:** 10.3390/metabo10030114

**Published:** 2020-03-19

**Authors:** Vuanghao Lim, Sara Ghorbani Gorji, Venea Dara Daygon, Melissa Fitzgerald

**Affiliations:** 1 School of Agriculture and Food Sciences, The University of Queensland, Brisbane, QLD 4072, Australia; s.ghorbanigorji@uq.edu.au (S.G.G.); v.daygon@uq.edu.au (V.D.D.); 2 Integrative Medicine Cluster, Advanced Medical and Dental Institute, Universiti Sains Malaysia, Bertam, Kepala Batas 13200, Penang, Malaysia

**Keywords:** Davidson’s plum, finger lime, native pepperberry, antioxidant, amino acids, metabolomics, GC×GC-TOFMS, UHPLC-QqQ-TOF-MS/MS, bush fruit

## Abstract

Selected Australian native fruits such as Davidson’s plum, finger lime and native pepperberry have been reported to demonstrate potent antioxidant activity. However, comprehensive metabolite profiling of these fruits is limited, therefore the compounds responsible are unknown, and further, the compounds of nutritional value in these native fruits are yet to be described. In this study, untargeted and targeted metabolomics were conducted using the three fruits, together with assays to determine their antioxidant activities. The results demonstrate that targeted free and hydrolysed protein amino acids exhibited high amounts of essential amino acids. Similarly, important minerals like potassium were detected in the fruit samples. In antioxidant activity, Davidson’s plum reported the highest activity in ferric reducing power (FRAP), finger lime in antioxidant capacity (ABTS), and native pepperberry in free radical scavenging (DPPH) and phosphomolybdenum assay. The compounds responsible for the antioxidant activity were tentatively identified using untargeted GC×GC-TOFMS and UHPLC-QqQ-TOF-MS/MS metabolomics. A clear discrimination into three clusters of fruits was observed using principal component analysis (PCA) and partial least squares (PLS) analysis. The correlation study identified a number of compounds that provide the antioxidant activities. GC×GC-TOFMS detected potent aroma compounds of limonene, furfural, and 1-R-α-pinene. Based on the untargeted and targeted metabolomics, and antioxidant assays, the nutritional potential of these Australian bush fruits is considerable and supports these indigenous fruits in the nutraceutical industry as well as functional ingredients for the food industry, with such outcomes benefiting Indigenous Australian communities.

## 1. Introduction

Australia is famous for its rich diversity of native plant foods, which are also known as bush tucker, or bush food. There are about 6500 types of bush foods, and only a handful have been commercialised, and are considered to be worth about $18–25 million to the Australian economy [1,2]. Among these, there are about 2400 native fruits found in Queensland alone. In the stocktake published by the Australian native food industry, [joint collaboration of the Australian Native Food Industry Limited (ANFIL) and Rural Industries Research and Development Corporation (RIRDC) now AgriFutures Australia], emphasis has focussed on twelve key crops for further development. In the list, several native fruits have been identified, such as Davidson’s plum, desert limes, quandong, lemon aspen, riberry, muntries, finger lime, kakadu plum, and native pepperberry [3]. These native edible fruits possess health benefits and can be used in applications such as functional foods and nutraceuticals, contributing to the emerging commercialisation in pharmaceutical industries. In the present work, three fruits were selected for in depth metabolomic profiling based on future potential: Davidson’s plum, native pepperberry, and finger lime.

Davidson’s plum (*Davidsonia pruriens* F. Muell) belongs to the Davidsoniaceae family, and it grows mostly in north-east Queensland in areas like coastal and upland rainforests (Djirbalngan, Yidinjdji, Djabuganjdji, Kuku-yalanji Nations). There are two other varieties; *D. johnsonii,* which is found in the south-east Queensland and New South Wales (Bundjalung Nation) while *D. jerseyana,* cultivates in the northern New South Wales (Bundjalung and Gumbainggir Nations) [4]. The tree can reach up to 20 m high [5]. The fruit is a purple plum described as tasting intensely sour [6], due to a high amount of acid with very little sugar to counteract it [6]. The fruit is rich in flavonoids, vitamins, minerals and several other important secondary metabolites, such as anthocyanins as well as proanthocyanidins [4]. Few preliminary studies have proclaimed that the extracts of Davidson’s plum fruit inhibited in vitro cancer cells, metabolic syndrome enzymes, and contained high antioxidant activity [7]. Belonging to the Rutaceae family, finger lime, *Citrus australasica* var. *sanguinea* is also called Rainforest Pearl, found in the rainforests of Queensland (Bundjalung Nation) and northern part of New South Wales (Gumbainggir Nation) [8,9]. The fruits come in various shapes and sizes, and a range of colours including purple, green, yellow and pink. The native finger lime cultivated in Australia is one of the seven citrus species with ‘caviar like’ appearance of the fruit pulp. In general, finger lime is rich in vitamins, minerals and terpenes, such as limonene [10]. The bioactivities and phytoconstituents of finger lime are not well-established with reported properties limited to antioxidant and anti-inflammatory activity [9,11]. Native pepperberry [Tasmannia lanceolata (Poir.) A.C. Smith], which belongs to the family of Winteraceae grows at highland areas in Tasmania (Palawa Nation) and southeastern Australia (Boonwurrung, Woiworung, Jaitmatang, Bidwell, Yuin, Ngarigo Nations). As a native shrub tree, the fruit is black (dark purple) in colour and contains many tiny black seeds [12]. Native pepperberry has been ethnopharmacologically used as an ailment to treat stomach discomfort, and as an antifungal for skin diseases by Indigenous people, and it has scientifically proven to inhibit in vitro platelet aggregation, microbial activity, as well as antioxidant capacity [13,14,15]. Sesquiterpene polygodial, a major phytoconstituent which is mostly found in the oil of native pepperberry makes the spicy and pungent flavour [14]. Additionally, other major secondary metabolites like guaiol, calamenene, hexacosanal, drimenol and linalool were also reported to possess antimicrobial properties [12,16].

In order to support the medical, nutritional and food significance of these bushfoods, it is important to identify the functional compounds and understand their activities. In this regard, the identification of phytoconstituents from various analyses is emphasised, considering the synergistic or antagonistic activity of the metabolite-metabolite interactions for certain bioactivities. Plant metabolomics provides the tools necessary to analyse and potentially identify all the metabolites that possess bioactive properties. Analytical platforms, such as ultra-high performance liquid chromatography mass spectrometry (UHPLC-MS), have been widely used in plant science for metabolomics applications to identify and quantify compounds [17,18]. The comprehensive profiling and metabolomics studies of bush fruits are important in reaping better insight into commercial viability of these fruits. Antioxidant activity has been reported for the fruits [7,11,14,15,19], however the correlation of the activity with bioactive compounds through metabolomics approaches has not been reported. Therefore, this study was conducted to bridge the gap for the discovery of antioxidant-based active metabolites together with comprehensive profiling of Davidson’s plum, finger lime and native pepperberry. In the current study, profiling of the fruits was conducted using targeted applications such as mineral analysis and amino acid analysis, and untargeted applications for semi-polar and aromatic compounds. Multivariate data analysis (MVDA), both supervised and un-supervised was applied to assess the association and discrimination of the compounds in the fruit samples. Data comparison of antioxidant scavenging activities was carried out together with total phenolic, flavonoids and flavonols content. The correlation between identified compounds and antioxidant activity was then conducted to identify potential bioactive markers in the fruit samples, as a mean of comprehensive findings for the fruits to be used in the nutraceutical industry.

## 2. Results

### 2.1. Untargeted Metabolic Profiling Using GC×GC-TOFMS and UHPLC-QqQ-TOF-MS/MS

**Figure 1:**
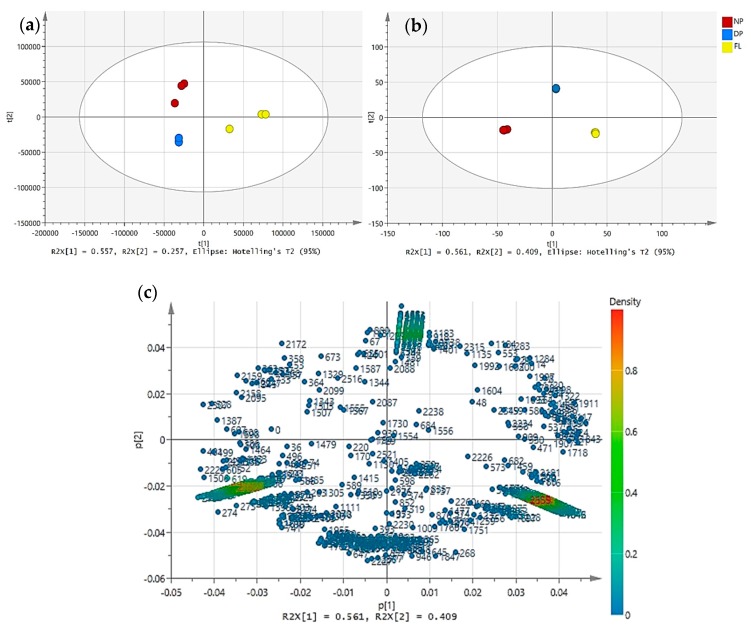
The principal component analysis of Davidson’s plum (DP), finger lime (FL) and native pepperberry (NP) using (**a**) GC×GX-TOFMS; (**b**) UHPLC-QqQ-TOF-MS/MS; and (**c**) loading score plot representing compounds using UHPLC-QqQ-TOF-MS/MS. Compounds are coloured to indicate the relative density of peak areas.

The aromatic compounds in the powdered fruits were analysed by two-dimensional gas chromatography time of fight mass spectrometry (GC × GC-TOFMS), and approximately 616 peaks were found from each sample. The samples were screened using ChromaTOF software for the presence of common components. A total of 604 compounds were tentatively identified in the samples of fruits based on the library match searching data in NIST 11 v 2.0 and our in-house library. The metabolite profiles were compared in the PCA scores plots by submitting the combined Davidson’s plum, finger lime and native pepperberry triplicate samples as shown in Figure 1a. Clustering of the scores was observed in three groups based on the three types of fruits. Two PCs were found with the greatest eigenvalues recorded at 55.7% and 25.7% of the total variance. The discrimination of the samples in three clusters indicates the aroma components in these fruits were different.

The untargeted metabolic profile of the fruit samples obtained from the negative mode of ultrahigh-performance liquid chromatography triple quadrupole-time-of-flight mass spectrometry/mass spectrometry (UHPLC-QqQ-TOF-MS/MS) provided 1166 compounds (after data processing), with 542 annotated compounds tentatively identified by MS1 and MS2 matchings; 478 by MS1 and 64 by MS2 matching only. The PCA analysis dataset of the 3 types of fruits provided distinct differences of clustering patterns as shown in Figure 1b. Noticeably, PC1 indicated 56.1% deviation between the 3 fruits with finger lime clustering on the right side, whereas native pepperberry was observed towards the negative quadrant of PC1. The PCA was further analysed in loading plot to observe the discrimination of the compounds (Figure 1c).

### 2.2. Antioxidant Activity Using DPPH, ABTS, FRAP and Phosphomolybdenum Assays

The fruit samples were subjected to in vitro antioxidant activity using different spectrophotometric assays as shown in Table 1. From the table, finger lime exhibited the strongest capability in ABTS, however the lowest in DPPH assay despite the same radical scavenging activity. In reducing power ability, Davidson’s plum reported the highest, while finger lime and native pepperberry did not show any significant difference. Phosphomolybdate method was later investigated for total antioxidant activity and expressed as gallic acid equivalent (µmol/gDW). The activity was found to decrease in the order of native pepperberry > finger lime > Davidson’s plum.

### 2.3. Total Phenolic Content (TPC), Total Flavonoid Content (TFC) and Total Flavonol Content (TFlC)

Further investigation was conducted for TPC, TFC and TFlC. Native pepperberry exhibited a significantly higher amount of TPC, TFC and TFlC compared to finger lime, as shown in Table 1. Finger lime showed the lowest gallic acid and quercetin equivalent for all the assays. These assays showed a similar trend for the samples, with native pepperberry the highest, followed by Davidson’s plum, and then finger lime.

### 2.4. Correlation Between Antioxidant Activity And Compounds in The GCMS Dataset

A supervised multivariate data, partial least squares (PLS) was applied to fathom the relationship between antioxidant activity and the fruit extracts. Based on Figure S1 in Supplementary Materials, the model showed cumulative R^2^X = 0.864 and Q^2^ = 0.886, indicating good fitness and high predictability (>0.5). In this analysis, the X-variables denote the aroma compounds and Y-variables are the antioxdant activity. Both PC1 and PC2 explained 86.4% of the variation in the aroma compounds showing discrimination in the compounds for antioxidant activity, with Y-variables recorded at 98%. In order to determine the aroma compounds that may contribute to the antioxidant activity, variable importance in projection (VIP) was carried out. The potential bioactive aroma compounds were chosen from the variables with VIP values of greater than 2.5. A total of 21 aroma compounds were sorted and the details are tabulated in Table 2. The identified aroma compounds were categorised into 6 different groups, namely, terpenes, aldehydes, terpenoids, furans, isoprenoids, and alkanes.

### 2.5. Correlation Between Antioxidant Activity And Compounds in The LCMS Dataset

**Figure 2:**
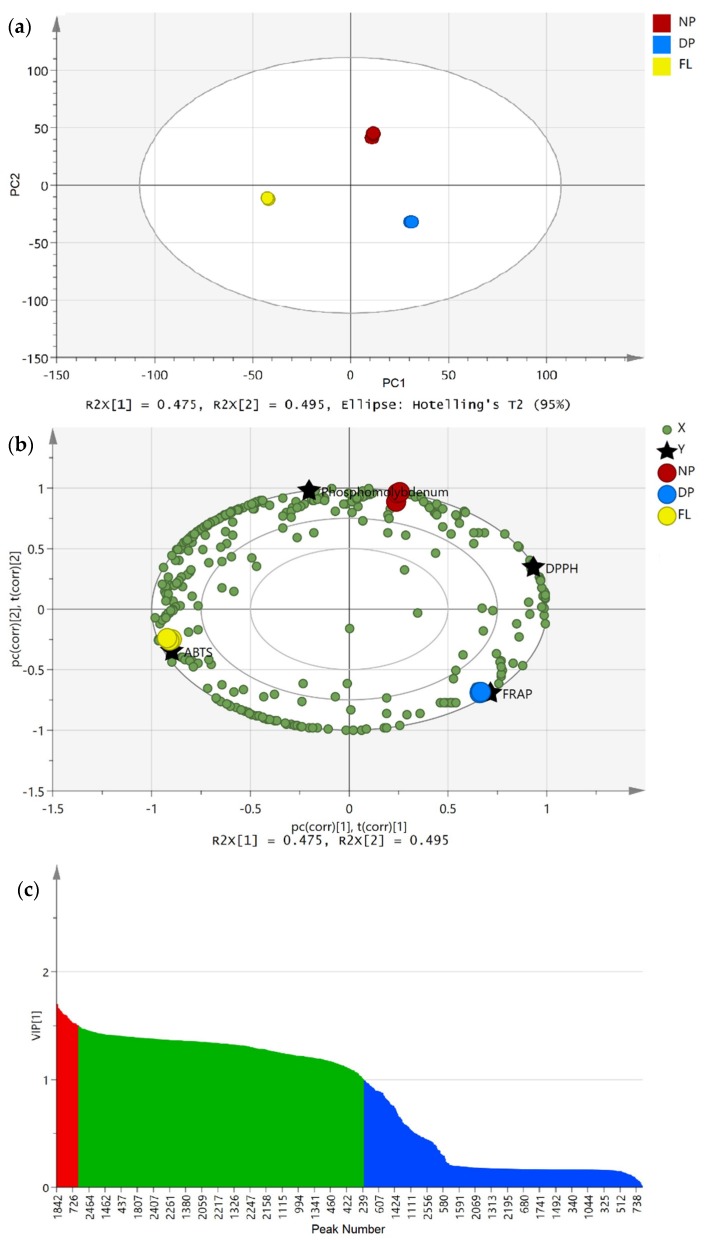
(**a**) Partial least square (PLS) score plot derived from UHPLC-QqQ-TOF-MS/MS on 3 fruit sample. (**b**) PLS biplot plots showing correlation between identified compounds with antioxidant activity. X = compounds, Y = antioxidant activity. (**c**) The variable importance in the projection (VIP) values (>1.5) represented by red coloured bars, (1.0–1.5) in green while blue coloured bars are VIP values (<1.0). The coloured compounds in (**b**) are VIP values >1.5.

With regard to the clear variance defined by the PCA (Figure 1b) and antioxidant activity (Table 2), a supervised multivariate data analysis, i.e., partial least squares (PLS) was utilised to organise and distinguish the samples according to their MS dataset. The correlation of the antioxidant activity and identified compounds was conducted by setting ABTS, DPPH, FRAP and phosphomolybdenum assays as Y variables, while the identified compounds were assigned as the X variables (Figure 2). In this experiment, a clear discrimination was achieved between Davidson’s plum, finger lime and native pepperberry with good discriminant model indicators, R^2^Y at 0.968 with the goodness-of-prediction value, Q^2^ at 0.965. The robustness of the PLS model on the antioxidant activity for discriminating the identified compounds was confirmed by the results of permutation tests (Figure S2 in Supplementary Materials). The correlation of identified compounds with antioxidant acitivty showed discrimination of the fruits with scattered Y variables as shown in Figure 2b. Y variables of DPPH and FRAP are seen in the right quadrant of PC1 (X = 47.5%; Y = 55.1%), whereas the ABTS and phosphomolybdenum assays are towards the left. PC2 accounts for 49.5% of X variables and 43.3% in Y variables.

Compounds that are responsible for the separation in Figure 2b were observed through variable importance in projection (VIP) selection approach. In this model, we set the VIP values at greater than 1.5 and loadings correlation coefficient [p(corr)] values above 0.5 to be significant for sample separation in the PLS model. From the analysis, a total of 44 compounds displayed good VIP values as listed in Table 3, with 7 unknown compounds. From the VIP scores, sugars, flavonoids and terpenes were the most important classes of compounds for antioxidant activity, followed by other phenolic compounds. Next, in order to better understand the distinctive incidence in antioxidant activity, we provide a fold-change analysis on the compounds (LogFC, Table 3). This analysis aims to correlate the significant value changes between the two group means. In this analysis, the fold-change was set at two, and any numbers that surpassed the threshold were considered significant. In particular, finger lime and native pepperberry showed the highest series of fold change with the fold-change distributions ranging from 3 to 10 times higher compared to Davidson’s plum and finger lime. Nevertheless, Davidson’s plum and native pepperberry only exhibited 5 significant fold-change of putatively identified compounds, ie. isovitexin, quercetagetin, racemosic acid, quercetin 3-[rhamnosyl-(1->2)-alpha-l-arabinopyranoside], and {3-[2-(3-hydroxy-5-methoxyphenyl)ethyl]phenyl}oxidanesulfonic acid, with the last compound also exhibited the highest fold-change (17.55).

### 2.6. Targeted Free And Protein Amino Acid Profilling Using UHPLC-MS

In total, 11 free amino acids were found in Davidson’s plum, 19 in finger lime and 14 in native pepperberry (Table 4). Interestingly, essential amino acid, lysine, exhibited the highest free amino acid for all the three fruit samples with Davidson’s plum (74.11%), finger lime (53.74%), and native pepperberry (67.88%). Other amino acids such as isoleucine, cystine and histidine are relatively high among the samples. Most of the free amino acids were detected in finger lime, and included aromatic amino acids, phenylalanine, tyrosine and tryptophan; sulfur-containing amino acids, cysteic acid, taurine and cystine; and non-essential amino acids, arginine, aspartic acid, glutamic acid, glycine, proline, and serine, but not alanine. Trace amounts of some free amino acids (<0.7%) were detected in native pepperberry compared to lysine, and isoleucine.

Analysis of the protein amino acids showed that Davidson’s plum exhibited 10 amino acids, and both finger lime and native pepperberry reported all the protein amino acids (Table 4). Similar to the profile of free amino acids, the three fruit samples demonstrated that lysine was the highest amount of hydrolysed protein amino acid, followed by isoleucine indicating a significant difference between the samples for isoleucine. lysine is significantly higher in Davidson’s plum compared to both finger lime and native pepperberry, in which the content is insignificant. Different trends of amino acids were observed for the three samples, where the percentage of amino acids in Davidson’s plum ranged from 55.53% (lysine) to 0.08% (valine), finger lime, from 45.185% (lysine) to 0.235% (norleucine), and native pepperberry, from 37.07% (lysine) to 0.18% (cysteic acid).

Un-supervised, PCA of the free and hydrolyed protein amino acids was later conducted to observe the association of the amino acids with the fruits (Figure S3 in Supplementary Materials). From the biplots, finger lime (positive PC1) is discriminated from Davidson’s plum and native pepperberry (negative PC1) and most of the free amino acids are associated with finger lime. In contrast, hydrolysed protein amino acids showed clustering of finger lime with native pepperberry and separating from Davidson’s plum with negative PC1. Almost all the amino acids were associated with finger lime and native pepperberry except lysine, cysteine and isoleucine.

### 2.7. Targeted Minerals and Heavy Metals Profilling using ICP-OES

**Figure 3:**
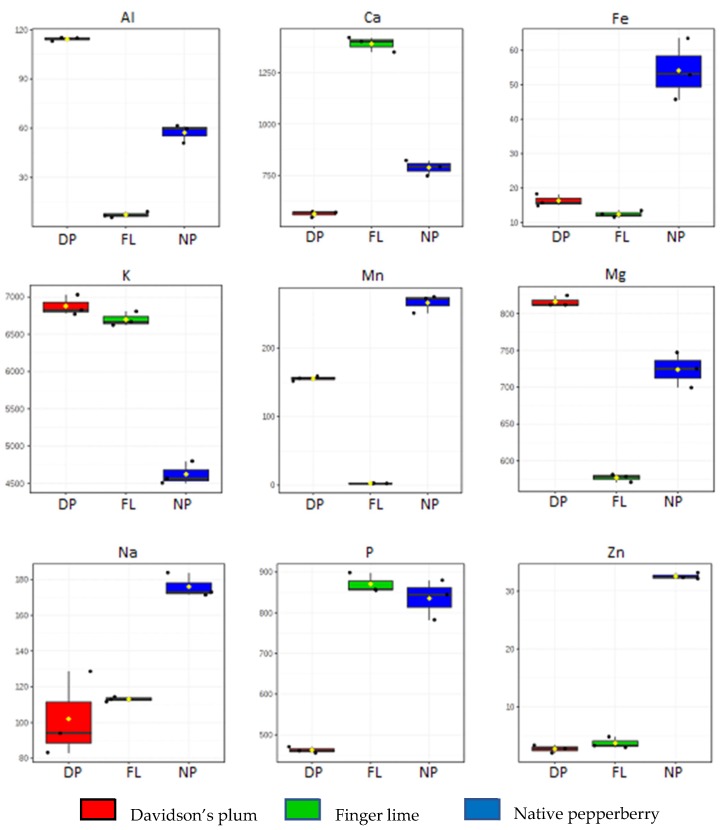
Box and whisker plots of nine elements varied significantly. DP: Davidson’s plum, FL: Finger lime, NP: Native pepperberry. The Y-axis of box and whisker plots indicates the amount in mg/kg.

Here, we targeted 18 minerals and heavy metals for the fruit samples and the analysed data are shown in Figure S4 in Supplementary Materials. The heat map shows the relative concentration of all the minerals and heavy metals in each sample. Minerals (Mn, Na, Fe, Zn) are abundantly distributed in native pepperberry compared to Davidson’s plum and finger lime. However, Davidson’s plum contained elevated levels of Mg (816.1 ± 7.4 mg/kg) and Al (114.2 ± 1.2 mg/kg) while finger lime with higher levels of Ca (1390.1 ± 35.8 mg/kg) and P (870.63 ± 24.2 mg/kg) than any other elements analysed in the study (Table S1 in Supplementary Materials). From the statistical analysis, 9 elements varied significantly (*p* < 0.05), i.e., Al, Ca, Fe, K, Mg, Mn, Na, P, and Zn as shown in Figure 3 and represented in box and whiskers plot. Interestingly, the relative amount of Al, K, and Mg were highest in Davidson’s plum compared to native pepperberry and finger lime. Al, Ca, Mg and P were moderately high in native pepperberry except for potassium. Finger lime exhibited a relatively low amount of most elements, but did show an amount of Ca and P compared to the other fruits.

## 3. Discussion

Davidson’s plum, finger lime and native pepperberry are among Australian native foods that are important to be classified as functional foods due to vast biologically active primary and secondary metabolites, especially terpenes, and flavonoids. Recently, with the inclusion of Indigenous foods in the food industry, natural antioxidants from bush fruits have gained expanding attention due to the growing demand for novel flavours, new functional compounds, and clean labelling. These fruits are rich in polyphenolic compounds, which are prominent natural antioxidants. The identification of aroma compounds using GC×GC-TOFMS provides an important factor in discriminating the clusters of the fruit samples. Correlation between antioxidant activity and aroma compounds exhibited abundance in terpenoid and terpene groups such as limonene, which generally contributes to the fruity smell due to its low odour threshold. Similar with previous studies, limonene was relatively high in finger lime, showing one of the major volatile compounds compared to other terpene groups [2,9,10]. The abundance of terpenes and terpenoids in the fruit samples suggest that they are suitable for processing of the fruits into jam and chutneys or even other food products with pleasant aroma and colour [9].

In the current study, we also conducted a correlation between bioactive compounds with antioxidant activity of the fruits using LCMS metabolomics. Through the comprehensive metabolite profiling, the results of various scavenging activities along with TPC, TFC and TFlC are presented in Table 2. In relation to the reported studies, Davidson’s plum exhibited proportionate amount of total phenolic content with damson plum, *Prunus domestica* subsp. *Insititia* L. (124.32 GAE µmol/gDW) cultivated in France [21], but three times higher (38.20 GAE µmol/gDW) compared with the same plum planted in Serbia [22]. In general, bush fruits are rich in phenolics, terpenes and flavonoids, forming the vital class of compounds for scavenging activities in human body [14]. Each antioxidant assay provides different data, therefore four types of antioxidant assays were conducted in order to completely evaluate the efficacy of the powdered fruit extracts. Generally, different fruits exhibited different strengths in their respective antioxidant assays when compared with gallic acid. The antioxidant capacity changes with in vitro assays depending on the affinities of the active compounds present in the fruits [23]. The outcome of this study is also synergistic with the TPC, TFC and TFlC activities with their respective antioxidant activity. Thus, these findings support the antioxidant function of the fruits, which is in good agreement with previous results [2,11,14,24,25].

The relative variability of the compounds in the fruits was measured using MVDA to characterise the tentative identification of peaks that are bio-actively related to antioxidant activity. PCA and PLS were employed with clear discrimination of the score plots among the fruits. The correlation of the compounds with antioxidant activity (DPPH, ABTS, FRAP and phosphomolybdenum) was conducted using a PLS biplot to reveal the dominant compounds that may be responsible for the activities. The search of the compounds that attributed to antioxidant activity was conducted through the VIP values greater than 1.5 (Table 3). Therefore, from the findings of both GC and LC chromatographic analyses, the identified notable compounds were highly possible for the antioxidant activity. Some aroma compounds such as limonene, furfural, α-pinene, terpinen-4-ol, and γ-terpinene and polar compounds like dicaffeoylquinic acid, quercetagetin, and 2′′-O-acetylrutin have been reported to possess potent antioxidant activity [22,23,24,25,26,27,28,29,30,31,32].

In addition to the secondary compounds identified, the fruits, especially finger lime and native pepperberry, are rich in amino acids. Nevertheless, most of the essential amino acids were present in the samples and can therefore act as a functional food in dietary supplements [33]. Though lysine is well represented in some vegetable species [34,35], it is absent in many plants compared to other amino acids and this is a disadvantage for vegans. According to the Academy of Nutrition and Dietetics (AND), vegetarians are recommended to consume an array of plant-based food that are rich in protein in order to meet nutritional and health requirements [36]. Interestingly, lysine exhibited the highest amount in the fruits, which is suitable for vegetarians as well as those who are allergic to beans and legumes [37].

Minerals and trace elements in fruits are acquired and transported naturally for biological processes in the plants. However, some of these minerals this could be toxic to humans when they occur at particular levels. Instead of posing a threat, mineral nutrients under the normal level assist in mitigating toxicity caused by heavy metals. In our mineral study, the ANOVA statistical analysis highlighted nine important minerals from the heatmap (Figure S4 in Supplementary Materials) of the fruit samples. As the major mineral in human dietary intake, potassium contributed the highest percentage for all the samples ranging from 52–68% showing a good source of potassium, especially for Davidson’s plum (68%). The requirement of potassium by humans is greater than 100 mg/day and with this fact, it is anticipated that the contribution of these fruits to dietary intake will grow in future. The potassium levels of Davidson’s plum (6877 ± 138 mg/kg), and finger lime (6697 ± 98 mg/kg) found in this study are higher than potassium levels in any Colombian fruits [38], and subtropical fruits grown in Spain, such as custard apple, avocado, mango, banana, papaya, persimmon and starfruit [39]. The amount of phosphorus (835 ± 49 mg/kg), calcium (788 ± 37 mg/kg) and magnesium (723 ± 23 mg/kg) were relatively high in native pepperberry, compared to jujube fruits grown in China [40]. Bush fruits have been reported for rich sources of calcium and magnesium with an added advantage because both minerals are important in forming DNA and are especially important in repairing damaged DNA. They are equally crucial in segregating the chromosomes during DNA synthesis [19].

Taken together, all the data indicate that these three native fruits are excellent sources of important compounds, that offer nutritional value to consumers. For the food industry, these fruits offer potential to extend shelf-life naturally, and increase the functional value of the food, enabling the industry to make health claims about the food. This information could also assist with the development of Indigenous businesses to supply these high value foods to the food industry. In addition, this study illustrates that the reason for different antioxidants values from the different methods is based on the nature and classes of the secondary compounds much more than any of the other compounds.

## 4. Materials and Methods

### 4.1. Chemicals and Reagents

All organic solvents were purchased from Fisher Scientific (Pittsburgh, PA, USA) unless stated otherwise. Ammonium ferrous sulfate, sulfuric acid, aluminium chloride, sodium nitrate, sodium hydroxide, sodium acetate, aluminium hexahydrate, ammonium molybdate, sodium phosphate, sodium nitrite, quercetin, gallic acid, glacial acetic acid, 2,4,6-Tris(2-pyridyl)-s-triazine (TPTZ), 2,2-Di(4-tert-octylphenyl)-1-picrylhydrazyl (DPPH), potassium persulfate, formic acid, 2,2′′-azinobis (3-ethylbenzothiazoline-6-sulfonic acid) diammonium salt (ABTS), were purchased from Sigma-Aldrich (St. Louis, MO, USA).

### 4.2. Plant Materials

Davidson’s plum, native pepperberry, and finger lime and were purchased from Taste Australia Bush Food Shop (Queensland, Australia). The samples were pulverised into fine powder using TissueLyser II (Qiagen, Tokyo, Japan). The pulverised samples were extracted with solvent or water (specified in respective method) in triplicates for chemical assays.

### 4.3. GC×GC-TOFMS Analysis

The aroma in fruits was comprehensively analysed using static headspace extraction coupled with separation by two-dimensional gas chromatography time-of-flight mass spectrometry (GC × GC-TOFMS, LECO Pegasus 4D, Castle Hill, Australia). Briefly, sample (500 mg) was weighed into 20 mL silicon capped GC headspace vials (Restek, Germany) and kept at −80 °C until further analysis. 2.5 mL headspace syringe was used to collect 1.5 mL of sample headspace after sample agitation of 10 min at 80 °C. An empty vial was used as blank and quality assurance (QA) standard was prepared by mixing all the samples. The program settings, conditions and parameters are provided in Table S2 in Supplementary Materials [41]. LECO ChromaTOF 4.50 software was used to process the GC×GC-TOFMS data for pre-processing baseline correction and identification was conducted by library matching (NIST 11 v2.0) and from authentic reference standards created in an in-house library. The similarity of ≥80% with the NIST library was defined as putative identification (when standards were not available) [42,43,44].

### 4.4. UHPLC-QqQ-TOF-MS/MS Analysis

The pulverised fruits powder (10 mg) from 3 plant species were dissolved in methanol (600 µL) and vortexed for 15 min at 30 °C. The mixture was then centrifuged at 28,500× *g* for 15 min and an aliquot of each supernatant (100 µL) was transferred into new Eppendorf tubes. Millipore water was added in ratio 3:1 to the supernatant. The mixture was vortexed and filtered through a 0.2 µm PTFE filter into an autosampler vial. For pooled biological quality control (PBQC) samples, 5 µL of each sample was taken and vortexed for 1 min. For the blank, 100 µL of Millipore water was used. The UHPLC-QqQ-TOF-MS/MS analysis was performed using Shimadzu Nexera UHPLC system (Kyoto, Japan; LC-30AD pump, SIL-30AC autosampler and CTO-30A column oven) equipped with Shimadzu Q-TOFMS-9030 detector. Separation of the sample analytes was conducted on a Shimadzu Velox C18 (2.1 × 100 mm, 1.8 µm, part number 227-32007-03, Shimadzu, Kyoto, Japan). The mobile phase consisted of A (0.1% [*v*/*v*] formic acid in acetonitrile) and B (0.1% [*v*/*v*] formic acid in water). The flow of the solvent gradient flow was set as follows: 97% A for 0–0.75 min, 5% A for 0.75–13 min, 97% A for 13–16 min. The sample injection volume was 1 µL with consistent flow rate at 0.4 mL/min. The column temperature and auto-sampler were set at 40 °C. Negative ionisation mode was operated for mass spectrometry analysis equipped with electrospray ionisation (ESI) source with collision energy set at 70 eV. The MS data were collected from *m*/*z* 70–700 Da; nebulisation gas at 3 L/min; source temperature, 120 °C; and desolvation temperature, 200 °C. The MS data were centroided and acquired with Lab Solutions software version 5.80. The raw data files were exported to Lab Solutions Insight software in LCD (*.lcd) format for pre-processing, correction of retention time, and baseline. The raw files were also converted to mzML format for peak discrimination, filtering and alignment using MS-DIAL [45,46].

### 4.5. Targeted Analysis of Free Amino Acids

#### 4.5.1. Extraction of Free Amino Acids from Fruit Samples

The samples were weighed (20 mg) in Eppendorf tube. Methanol (500 µL) was added and vortexed. The mixture was centrifuged at 30,000× *g* for 5 min, then the supernatant was transferred into a 2 mL tube. The precipitate was resuspended in 500 µL of Millipore water and vortexed. Next, the mixture was centrifuged at the same speed for 5 min. The methanol and water extract were combined and filtered through a 0.2 µm PTFE filter.

#### 4.5.2. Free Amino Acid Derivatisation

The free amino acids were derivatised using AccQ.Fluor^TM^ reagent according to the Waters AccQ.Tag^TM^ pre-column derivatisation procedure [47]. Briefly, 35 µL of borate buffer was put into a tube (2 mL). 5 µL of sample was then mixed and vortexed for several seconds. Ten µL of reconstituted derivatisation reagent were then admixed to the buffered samples and immediately vortexed. Next, the mixture was left at room temperature for 1 min. After that, the samples were heated in a heating block for 10 min at 55 °C to finalise the derivatisation. The derivatised free amino acids were then transferred to autosampler vial for analysis. PBQC samples were prepared by pooling 5 µL from each sample, vortexed for 1 min and derivatised using the same method as mentioned previously.

#### 4.5.3. Standards

Standard amino acid mix solution (Batch. No SLBS2232V; Sigma-Aldrich, St. Louis, MO, USA) was used for the identification and quantification of amino acids. The standards were prepared in serial dilutions of 40% stock solutions to 20%, 10%, 5%, 2.5% and 1.25%. Derivatisation of the standard amino acids were conducted according to the methods explained in Section 4.5.2. As for blank, 5 µL of Millipore water was used to replace sample at the beginning step, then followed by the derivatisation steps mentioned in Section 4.5.2.

#### 4.5.4. UHPLC-MS Conditions

The analysis of amino acid derivatives was analysed on a Shimadzu Nexera UHPLC system (Kyoto, Japan; LC-30AD pump, SIL-30AC autosampler and CTO-30A column oven) equipped with Shimadzu MS-2020 detector. Waters Acquity UPLC^TM^ BEH C_18_ column (2.1 × 100 mm, 1.7 µm, part number 186003837, Waters, Milford, MA, USA) was used for the chromatographic separation at consistent temperature of 55 °C. The MS parameters were set as follows: Acquisition mode, SIM (refer Table S3 in Supplementary Materials); detector voltage, 0.1 V; interface voltage, 2.5 V; ionisation mode, positive; heat block temperature, 500 °C; interface temperature, 350 °C; nebulising gas flow, 1.5 L/min; injection volume, 10 µL. The mobile phase consists of A: 0.1% formic acid (*v*/*v*) in Millipore water, and B: 0.1% formic acid (*v*/*v*) in acetonitrile. The flow rate was set at 0.7 mL/min based on the gradient profile: initial-0.54 min (0–0.1% B); 0.54–5.74 min (0.1–15% B); 5.74–8.74 min (15–21.2% B); 8.74–10.50 min (21.2–59.6% B); 10.50–11.50 min (59.6% B); 11.50–12.00 min (59.6–0.1% B) and finally at 0.1% B until 13 min. The interconnected cleaning purge was set within 1 min (rinsing speed 35 µL/sec), and equilibrium was repeated for 5 min at initial conditions. The whole cycle time took 13 min to complete before the next injection.

### 4.6. Targeted Analysis of Protein Amino Acids

#### 4.6.1. Sample Digestion

Fruit samples (20 mg each pulverised) were digested in a glass vessel containing 2 mL of 6 N HCl and 0.1% of phenol. The glass vessels were flushed with N_2_ before sealing. Samples were hydrolysed at 110 °C for 20 h. Next, the samples were filtered using 0.2-µm filter, and the filtrate was neutralised with freshly prepared 6 N NaOH solution [48].

#### 4.6.2. Protein Amino Acid Derivatisation

The protein amino acid derivatisation, PBQC and standards were prepared according to the methods mentioned in Section 4.5.2 and Section 4.5.3.

#### 4.6.3. UHPLC-MS Analysis

The standards and derivatised protein amino acid were injected and analysed according to the UHPLC-MS conditions mentioned in Section 4.5.4.

### 4.7. Targeted Analysis of Minerals and Heavy Metals

#### 4.7.1. Sample Preparation

The fruits samples were weighed (100 mg) into new and clean 15 mL digestion tubes. To each tube, concentrated nitric acid (2.0 mL) was added and samples were pre-digested overnight. They were then placed into a digestion rack of Hotblock^®^ Digestor SC100 Digestion System (Environmental Express, Vernon Hills, IL, USA). The samples were digested for 1 h at 100 °C. Upon completion, the samples were cooled, and then water added to bring the volume to 15 mL. The samples were shaken and centrifuged for 5 min [49].

#### 4.7.2. ICP-OES Analysis

The operating conditions for inductively coupled plasma optical emission spectrometer (ICP-OES) were performed according to the parameters: RF incident power, 1000 W; plasma argon flow rate, 15.0 mL/min; auxillary argon flow rate, 1.50 mL/min; nebulizer argon flow rate, 0.75 mL/min; mist chamber, tracey and nebulizer SeaSpray, 2 mL/min flow rate. The wavelength measured for each element is as follows: Al 396.152 nm, As 188.980 nm, Ca 422.673 nm, Cd 214.439 nm, Co 238.892 nm, Cr 267.716 nm, Cu 324.754 nm, Fe 238.204 nm, K 766.491 nm, Mg 279.553 nm, Mn 257.610 nm, Mo 202.032 nm, Na 588.995 nm, Ni 231.604 nm, P 213.618 nm, Pb 220.353 nm, S 181.972 nm, and Zn 213.857 nm.

### 4.8. Determination of in Vitro Antioxidant Activity

The DPPH, FRAP, ABTS and phosphomolybdenum activities of the fruit samples were evaluated by the methods reported by [44,50,51], respectively. The ABTS, DPPH and phosphomolybdenum assays were reported as gallic acid equivalents (GAE µmol/gDW), whereas FRAP as Fe^2+^ equivalents (Fe^2+^ µmol/gDW).

### 4.9. Total Phenolic, Flavonoid and Flavonol Contents

Spectrophotometric technique was used for total phenolic, flavonoid and flavonol contents and were quantified following the protocol of [52,53]. Total phenolic contents were expressed as gallic acid equivalents (GAE µmol/gDW), whereas total flavonoid and flavonol contents were presented as quercetin equivalents (QTE µmol/gDW).

### 4.10. Data Processing and Analysis

Data for bioactivities were presented as mean ± SD for all triplicate analysis and a one-way analysis of the variance (ANOVA) was carried out using SPSS Statistics version 25 (IBM Corp, Armonk, NY, USA). The mean comparisons were conducted using the post-hoc Tukey’s (HSD) multiple comparison test. Values with *p* < 0.05 were considered statistically significant.

The processed data from GC×GC-TOFMS were analysed using SIMCA-P software version 15 (Umeå, Sweden) for multivariate data analysis (MVDA). In order to access the clustering and trends of the comprehensive depiction of the fruit samples, principal component analysis (PCA) was used. Partial least squares (PLS) chemometric method was conducted to further analyse the correlation of antioxidant activity with volatile compounds in the fruit samples [41,44]. The variables were pareto scaled for PCA and PLS analyses. Afterwards, the variables selection namely VIP was used to select those notable volatile compounds (VIP > 2.5).

For UHPLC-QqQ-TOF-MS/MS data, the variables were pareto scaled and log transformed for PCA and PLS to lower heteroscedasticity and asymmetry in the statistical distribution [44]. In order to tentatively identify the compounds, the acquired raw data in mzML format were processed using MS-DIAL by comparing the MS/MS spectra with those in the spectral library. VIP was applied to identify the most significant compounds contributing to the antioxidant activity [54]. Compounds with VIP score > 1.5 were maintained for further elaboration using methods from MetaboAnalyst 4.0, an open source web-based tools for metabolomics data analysis [55].

For targeted MVDA of amino acids, both targeted free and hydrolysed amino acids were pareto scaled and log transformed. The heatmap and box and whiskers were generated using MetaboAnalyst 4.0 [55].

## 5. Conclusions

We have successfully reported that the Australian bush fruits, Davidson’s plum, finger lime and native pepperberry are rich in terpenes, phenolics, flavonoids, flavonols, minerals, essential and non-essential free and hydrolysed protein amino acids, and functional bioactive compounds, which are promising fruits to be commercialised in the nutraceutical industry and food industry. We found that the fruits are an abundant source of antioxidant compounds (sugars, terpenes, flavonoids etc.) and that may serve as the source of natural antioxidants in food products or as new medicines. The use of metabolomics in correlation to the primary and secondary metabolites as well as the minerals would render an opportunity to obtain more potential bioactivities of the Australian bush fruits as functional food in nutraceutical industry. We also show that when reporting antioxidant activity, it is important to use more than one method to obtain a true understanding of the activity.

## Figures and Tables

**Table 1:** In vitro antioxidant activity of the 3 fruit samples. Data is expressed in dry weight, DW and presented as mean values ± standard deviation (*n* = 3). Different superscript letters within each column indicate significant (*p* < 0.01) difference between samples.

Samples	ABTS (GAEµmol/gDW)	DPPH (GAEµmol/gDW)	FRAP (µmol Fe^2+^/gDW)	Phosphomolybdenum (GAEµmol/gDW)	TPC (GAEµmol/gDW)	TFC (QTE µmol/gDW)	TFlC (QTE (µmol/gDW)
DP	21.92 ± 1.60 ^a^	97.38 ± 3.93 ^b^	500.38 ± 64.32 ^c^	52.71 ± 5.40 ^a^	113.58 ± 14.20 ^b^	11.31 ± 0.52 ^a^	5.55 ± 0.08 ^b^
FL	62.73 ± 0.55 ^c^	17.23 ± 2.04 ^a^	46.16 ± 3.74 ^a^	114.89 ± 3.25 ^b^	63.46 ± 1.10 ^a^	10.59 ± 0.97 ^a^	0.56 ± 0.18 ^a^
NP	22.06 ± 3.43 ^a^	119.49 ± 2.72 ^c^	48.48 ± 3.23 ^a^	291.94 ± 2.23 ^c^	134.82 ± 11.08 ^b^	35.86 ± 3.99 ^b^	9.99 ± 0.21 ^c^

ABTS: 2,2′-azino-bis(3-ethylbenzothiazoline-6-sulfonic acid); DPPH: 2,2-diphenyl-1-picrylhydrazyl; FRAP: Ferric Reducing of Antioxidant Power Assay; TPC: Total Phenolic Content; TFC: Total Flavonoid Content; TFlC: Total Flavonol Content; DP: Davidson’s plum; FL: Finger lime; NP: Native pepperberry; GA: Gallic acid equivalent; QTE: Quercetin equivalent.

**Table 2:** Aroma compounds identified by variable importance in projection (VIP) > 2.5 selection method for Davidson’s plum, finger lime and native pepperberry.

Peak No.	Compounds	Group (VIP Score)	Retention Time (1t_R,_ 2t_R_) ^a^	CAS No.	Mf ^b^	Exact Mass	Level ^c^
526	1-methyl-4-(prop-1-en-2-yl)cyclohex-1-ene [Limonene]	Terpene (7.01)	769.55, 0.72	138-86-3	C_10_H_16_	136.23	L2
329	1-R-α-Pinene	Terpene (6.34)	716.15, 637.29	7785-70-8	C_10_H_16_	136.23	L2
498	Furfural	Aldehyde (5.58)	577.36, 1.28	98-01-1	C_5_H_4_O_2_	96.08	L1
508	Hexanal	Aldehyde (5.12)	503.75, 0.69	66-25-1	C_6_H_12_O	100.15	L1
437	1-methyl-4-(1-methylethylidene)- cyclohexene [Terpinolene]	Terpenoid (4.74)	972.00, 0.98	586-62-9	C_10_H_16_	136.23	L2
410	γ-Terpinene	Terpene (4.70)	1072.61, 1.18	99-85-4	C_10_H_16_	136.23	L2
599	Terpinen-4-ol	Terpenoid (4.49)	1038.69, 1.00	562-74-3	C_10_H_18_O	154.24	L2
397	2,2-dimethyl-3-methylidenebicyclo[2.2.1]heptane [Camphene]	Terpenoid (4.28)	953.92, 1.06	79-92-5	C_10_H_16_	136.23	L2
326	α-Phellandrene	Terpene (3.78)	778.35, 0.70	99-83-2	C_10_H_16_	136.23	L2
342	1-methyl-4-(1-methylethenyl)-benzene [p-Cymenene]	Terpene (3.63)	960.45, 0.88	1195-32-0	C_10_H_12_	132.20	L2
493	2-ethylfuran	Furan (3.55)	367.27, 0.88	3208-16-0	C_6_H_8_O	96.12	L2
281	1-methyl-4-(1-methylethyl)-7-Oxabicyclo [2.2.1]heptane	Alkane (3.38)	757.76, 0.68	470-67-7	C_10_H_18_O	154.24	L2
1	(-)-Carvone	Terpenoid (3.11)	1170.77, 1.10	6485-40-1	C_10_H_14_O	150.21	L2
331	α-Terpineol	Terpenoid (3.05)	1072.61, 1.18	98-55-5	C_10_H_18_O	154.24	L2
527	l-menthone	Terpenoid (2.90)	1018.87, 0.87	10458-14-7	C_10_H_18_O	154.24	L2
364	4-methylene-1-(1-methylethyl)- Bicyclo[3.1.0]hexane, [Sabinene]	Terpene (2.89)	799.78, 0.70	3387-41-5	C_10_H_16_	136.23	L2
399	Caryophyllene	Isoprenoid (2.82)	1399.12, 0.71	87-44-5	C_15_H_24_	204.35	L2
569	Pentanal	Aldehyde (2.81)	382.87, 0.67	110-62-3	C_5_H_10_O	86.13	L2
272	5-isopropenyl-2-methylcyclopent-1-enecarboxaldehyde	Terpenoid (2.78)	1275.00, 0.95	3865-09-6	C_10_H_14_O	150.21	L2
271	4-methylene-5-hexenal	Aldehyde (2.52)	629.52, 0.90	17844-21-2	C_7_H_10_O	110.15	L2
469	Dodecane	Alkanes (2.51)	984.85, 0.61	112-40-3	C_12_H_26_	170.33	L1

^a^ 1t_R_: First dimension retention time, 2t_R_: Second dimension retention time; ^b^ Mf: Molecular formula; ^c^ Level: Level of identification based on the guidelines [20]. L1—level 1 identified through authentic chemical standards; L2—putatively identified compounds through library matching.

**Table 3:** PLS modelling of discriminant putatively identified compounds by variable importance in the projection (VIP) selection method based on antioxidant activity. The list of VIP scores are provided together with Log fold-change (LogFC) values.

No	Var ID	VIP Compounds	Class	VIP Score	p(corr)	LogFC [DP vs. FL]	LogFC [DP vs. NP]	LogFC [FL vs. NP]
1	1842	β-D-Glucuronopyranosyl-(1->3)-a-D-galacturonopyranosyl-(1->2)-L-rhamnose	Oligosaccharides	1.70	−9.67 × 10	−5.83	-	16.85
2	1845	Dicaffeoylquinic acid	Quinic acids and derivatives	1.70	−9.67 × 10^3^	−5.79	-	16.81
3	1846	Formononetin 7-(6′′-malonylglucoside)	Isoflavonoid O-glycosides	1.69	−9.67 × 10^3^	−5.73	-	16.75
4	2010	Octotiamine	Aminopyrimidines and derivatives	1.66	−9.67 × 10^3^	−4.83	-	15.85
5	268	Quercetagetin	Flavonoid-7-O-glycosides	1.66	−9.39 × 10^3^	−4.94	−7.61	8.36
6	2447	2′′-O-Acetylrutin	Flavonoid-3-O-glycosides	1.65	−9.67 × 10^3^	−4.69	-	15.71
7	2172	Quercetin 3-[rhamnosyl-(1->2)-alpha-L-arabinopyranoside]	Flavonoid-3-O-glycosides	1.64	9.92 × 10^3^	3.69	6.88	-7.83
8	820	Suaveolenine	3-alkylindoles	1.64	−9.67 × 10^3^	−4.43	-	15.44
9	1797	Kurzichalcolactone B	2′-Hydroxychalcones	1.63	−9.67 × 10^3^	−4.23	-	15.25
10	1789	Syringetin-3-O-glucoside	Flavonoid-3-O-glycosides	1.63	−9.67 × 10^3^	−4.16	-	15.18
11	310	3-(5-methoxy-2,2-dimethyl-1-benzopyran-8-yl)-3-oxopropanoic acid	2,2-dimethyl-1-benzopyrans	1.62	−9.67 × 10^3^	−4.07	-	15.09
12	1085	Bakkenolide D	Terpene lactones	1.62	−9.67 × 10^3^	−3.87	-	14.89
13	712	3-Feruloyl-1,5-quinolactone	Coumarins and derivatives	1.61	−9.67 × 10^3^	−3.81	-	14.83
14	1857	6-{7-Acetoxy-5-chloro-3-[(1E,3E)-3,5-dimethyl-1,3-heptadien-1-yl]-7-methyl-6,8-dioxo-7,8-dihydro-2(6H)-isoquinolinyl}norleucine	Amino acid derivatives	1.61	−9.67 × 10^3^	−3.64	-	14.66
15	1592	5,7-dihydroxy-2-(4-hydroxy-3-methoxyphenyl)-3-{[3,4,5-trihydroxy-6-(hydroxymethyl)oxan-2-yl]oxy}-4H-chromen-4-one	Flavonoid-3-O-glycosides	1.60	−9.67 × 10^3^	−3.49	-	14.51
16	1189	Unknown	-	1.60	−9.67 × 10^3^	−3.47	-	14.49
17	819	Unknown	-	1.60	−9.67 × 10^3^	−3.44	-	14.46
18	1188	farnochrol	Terpene lactones	1.59	−9.67 × 10^3^	−3.40	-	14.42
19	581	Perilloside B	Terpene glycosides	1.59	−9.66 × 10^3^	−3.37	-	14.39
20	1847	Racemosic acid	Phenolic glycosides	1.59	−8.95 × 10^3^	−4.28	−9.18	6.12
21	2341	Kaempferol 3-[2′′′-acetyl-alpha-L-arabinopyranosyl-(1->6)-galactoside]	Flavonoid-3-O-glycosides	1.58	−9.67 × 10^3^	−3.16	-	14.18
22	1851	Dracunculifoside G	Coumaric acids and derivatives	1.58	−9.66 × 10^3^	−3.12	-	14.14
23	583	Betuloside	Fatty acyl glycosides of mono- and disaccharides	1.57	−9.66 × 10^3^	−2.83	-	13.85
24	2404	Isosakuranetin-7-O-neohesperidoside	Flavanone	1.56	−9.66 × 10^3^	−2.76	-	13.78
25	2000	6-(benzoyloxy)-1-(hexopyranosyloxy)-1,4a,5,6,7,7a-hexahydro-5-hydroxy-7-methyl-cyclopenta[c]pyran-4-carboxylic acid	Carboxyl	1.56	−9.67 × 10^3^	−2.68	-	13.69
26	413	Unknown	-	1.55	−9.66 × 10^3^	−2.52	-	13.54
27	1304	Unknown	-	1.55	−9.67 × 10^3^	−2.35	-	13.37
28	1802	Pungiolide A	Xanthanolides	1.54	−9.67 × 10^3^	−2.28	-	13.30
29	817	Speradine A	Isoindolones	1.54	−9.67 × 10^3^	−2.25	-	13.27
30	1228	Isovitexin	Flavone	1.53	−9.49 × 10^3^	−1.85	−3.22	9.65
31	2016	6-methoxy-7-[3,4,5-trihydroxy-6-[(3,4,5-trihydroxy-6-methyloxan-2-yl)oxymethyl]oxan-2-yl]oxychromen-2-one	Ketone	1.53	−9.66 × 10^3^	−1.94	-	12.96
32	2388	Unknown	-	1.53	−9.66 × 10^3^	−1.90	-	12.92
33	726	4-Methylumbelliferyl glucuronide	Coumarins and derivatives	1.52	−9.67 × 10^3^	−1.77	-	12.78
34	1697	3,4,5-trihydroxy-6-{3,4,5-trihydroxy-2-[3-(4-methoxyphenyl)-2-oxopropanoyl]phenoxy}oxane-2-carboxylic acid	Flavonoid O-glycosides	1.52	−9.81 × 10^3^	−1.12	-	11.36
35	1949	Unknown	-	1.52	−9.67 × 10^3^	−1.70	-	12.72
36	582	3-Hydroxy-4-isopropylbenzyl alcohol 3-glucoside	Terpene glycosides	1.52	−9.66 × 10^3^	−1.70	-	12.72
37	2256	Unknown	-	1.52	−9.65 × 10^3^	−1.80	-	12.81
38	552	{3-[2-(3-hydroxy-5-methoxyphenyl)ethyl]phenyl}oxidanesulfonic acid	Stilbenes	1.52	7.05 × 10^3^	6.53	17.55	
39	1020	Picraquassioside A	Phenolic glycosides	1.52	−9.66 × 10^3^	−1.65	-	12.67
40	834	(2S)-2-Butanol O-[b-D-Apiofuranosyl-(1->6)-b-D-glucopyranoside]	O-glycosyl compounds	1.51	−9.67 × 10^3^	−1.62	-	12.64
41	1939	Longipedunin A	Hydrolyzable tannins	1.50	−9.66 × 10^3^	−1.43	-	12.45
42	441	10,11-epoxycurvularin	Aryl alkyl ketones	1.50	−9.67 × 10^3^	−1.36	-	12.38
43	727	Moschamine	N-acylserotonins	1.50	−9.67 × 10^3^	−1.34	-	12.36
44	2131	Unknown	-	1.50	−9.66 × 10^3^	−1.32	-	12.34

Var ID: Peak number; p(corr): correlation coefficient; DP: Davidson’s plum; FL: Finger lime; NP: Native pepperberry.

**Table 4:** Amino acid composition of Davidson’s plum (DP), finger lime (FL) and native pepperberry (NP) for (**a**) free amino acids; and (**b**) hydrolysed protein amino acids. Results are expressed as mean values (µmol/g) ± SD (*n* = 3). Superscript letters within each column indicate statistically significant (*p* < 0.05; Tukey test). ND = not detected.

(**a**)											
**Sample**	**Alanine**	**Arginine**	**Aspartic Acid**	**Cysteic Acid**	**Cystine**	**Glutamic Acid**	**Glycine**	**Histidine**	**Isoleucine**	**Leucine**	**Lysine**
DP	ND	0.16 ± 0.05 ^a^	0.04 ± 0.04 ^a^	ND	0.63 ± 0.15 ^a^	0.18 ± 0.16 ^a^	0.30 ± 0.03 ^b^	0.57 ± 0.05 ^a^	7.48 ± 0.71 ^a^	0.03 ± 0.01 ^a^	27.43 ± 0.96 ^a^
FL	ND	5.60 ± 0.35 ^b^	0.77 ± 0.09 ^b^	1.80 ± 0.12 ^a^	0.59 ± 0.12 ^a^	0.16 ± 0.14 ^a^	0.25 ± 0.04 ^b^	2.91 ± 0.10 ^b^	7.58 ± 1.13 ^a^	0.04 ± 0.01 ^a^	26.52 ± 1.62 ^a^
NP	ND	0.06 ± 0.007 ^a^	0.04 ± 0.003 ^a^	1.79 ± 0.11 ^a^	0.53 ± 0.21 ^a^	0.56 ± 0.28 ^a^	0.05 ± 0.02 ^a^	ND	8.20 ± 1.62 ^a^	0.04 ± 0.005 ^a^	25.18 ± 1.59 ^a^
**Sample**	**Methionine**	**Norleucine**	**Phenylalanine**	**Proline**	**Serine**	**Taurine**	**Threonine**	**Tryptophan**	**Tyrosine**	**Valine**	
DP	ND	0.11 ± 0.01 ^a^	0.08 ± 0.007 ^a^	ND	ND	ND	ND	ND	ND	ND	
FL	ND	0.11 ± 0.02 ^a^	0.14 ± 0.003 ^a^	0.50 ± 0.04 ^b^	0.45 ± 0.09	1.07 ± 0.01	0.19 ± 0.06	0.03 ± 0.02	0.36 ± 0.05	0.27 ± 0.07 ^a^	
NP	0.04 ± 0.01	0.10 ± 0.01 ^a^	0.08 ± 0.007 ^a^	0.22 ± 0.03 ^a^	ND	ND	ND	ND	ND	0.20 ± 0.18 ^a^	
(**b**)											
**Sample**	**Alanine**	**Arginine**	**Aspartic Acid**	**Cysteic Acid**	**Cystine**	**Glutamic Acid**	**Glycine**	**Histidine**	**Isoleucine**	**Leucine**	**Lysine**
DP	ND	ND	0.00 ± 0.006 ^a^	ND	0.39 ± 0.31 ^a^	0.46 ± 0.42 ^a^	ND	ND	17.62 ± 0.43 ^a^	0.34 ± 0.10 ^a^	66.22 ± 2.92 ^a^
FL	1.71 ± 0.78 ^a^	5.21 ± 1.75 ^a^	28.71 ± 2.17 ^c^	0.64 ± 0.56 ^a^	0.62 ± 0.04 ^a^	6.99 ± 2.32 ^b^	6.42 ± 2.88 ^a^	2.76 ± 1.03 ^a^	30.01 ± 2.19 ^b^	5.05 ± 1.41 ^b^	114.68 ± 16.62 ^b^
NP	9.72 ± 0.91 ^b^	4.25 ± 1.10 ^a^	13.38 ± 0.93 ^b^	0.60 ± 0.52 ^a^	0.61 ± 0.02 ^a^	36.58 ± 1.94 ^c^	11.93 ± 0.90 ^b^	3.70 ± 0.50 ^a^	28.45 ± 5.25 ^b^	14.70 ± 2.56 ^c^	117.28 ± 14.24 ^b^
**Sample**	**Methionine**	**Phenylalanine**	**Proline**	**Serine**	**Threonine**	**Tryptophan**	**Tyrosine**	**Valine**			
DP	ND	2.33 ± 0.27 ^b^	ND	ND	ND	16.18 ± 1.18 ^a^	15.24 ± 1.16 ^b^	0.10 ± 0.01 ^a^			
FL	1.07 ± 0.68 ^a^	1.48 ± 0.54 ^a^	8.45 ± 1.50 ^b^	3.66 ± 0.27 ^a^	6.80 ± 1.52 ^a^	15.29 ± 0.43 ^a^	12.90 ± 0.07 ^a^	0.74 ± 0.10 ^a^			
NP	5.10 ± 0.28 ^b^	4.11 ± 0.06 ^c^	1.83 ± 0.67 ^a^	12.61 ± 1.38 ^b^	6.52 ± 1.12 ^a^	15.48 ± 0.05 ^a^	13.97 ± 0.82 ^a^	14.13 ± 0.80 ^b^			

## Supplementary Materials

The following are available online at https://www.mdpi.com/2218-1989/10/3/114/s1, Table S1: Targeted analysis of 18 minerals and heavy metals from Davidson’s plum (DP), finger lime (FL) and native pepperberry (NP). Results are expressed as mean values (mg/DWkg) ± SD (*n* = 3). Superscript letters within each column indicate statistically significant (*p* < 0.05; Tukey test), Table S2: GC×GC-TOF-MS parameters for comprehensive profiling of fruit samples, Table S3: Amino acids’ single ion monitoring for mass spectrometry detector, Figure S1: (**a**) Partial least square (PLS) score plot based on GC×GX-TOFMS data. (**b**) PLS biplot plots showing correlation between identified aroma compounds with antioxidant activity. X = compounds, Y = antioxidant activity, Figure S2: Permutation test of the PLS model based on UHPLC-QqQ-TOF-MS/MS data (**a**) ABTS; (**b**) DPPH; (**c**) FRAP; (**d**) Phosphomolybdenum assays, Figure S3: Biplot association of (**a**) free amino acid; (**b**) hydrolysed protein amino acid in Davidson’s plum (DP), finger lime (FL) and native pepperberry (NP), Figure S4: Heat map of 18 mineral nutrients and heavy metals found in the fruit samples.
