# Supplementary material for: Untargeted and Targeted Metabolomic Profiling of Australian Indigenous Fruits
Source: Metabolites. 2020 Mar 19;10(3):114. doi: 10.3390/metabo10030114 (PMC7143387; doi:10.3390/metabo10030114)
Supplement: Supplementary file 1 [file metabolites-10-00114-s001.pdf]

Supplementary Materials

Table S1. Targeted analysis of 18 minerals and heavy metals from Davidson's plum (DP), finger lime (FL) and native pepperberry (NP). Results are expressed as mean values (mg/DWkg) ± SD (n=3). Superscript letters within each column indicate statistically significant ($p<0.05$; Tukey test).

Sample	Al	As	Ca	Cd	Co	Cr	Cu	Fe	K	Mg	Mn	Mo	Na	Ni	P	Pb	S	Zn
DP	114.2± 1.2^{a}	9.0± 4.9^{a}	563.3± 15.2^{c}	4.4± 0.5^{a}	1.8± 1.4^{a}	1.3± 0.8^{a}	9.0± 0.1^{a}	16.3± 1.7^{b}	6877.6± 138.0^{a}	816.1± 7.4^{a}	155.5± 3.4^{a}	0.0± 0.0^{a}	102.0± 23.8^{b}	1.5± 0.6^{a}	462.5± 7.6^{b}	1.0± 0.1^{a}	941.3± 15.8^{a}	2.8± 0.7^{b}
FP	7.2± 1.7^{c}	18.7± 6.9^{a}	1390.2± 35.8^{a}	1.7± 2.8^{a}	1.5± 0.6^{a}	1.2± 0.8^{a}	8.3± 0.5^{a}	12.4± 1.0^{b}	6697.4± 98.7^{a}	577.0± 5.4^{c}	2.6± 0.2^{c}	1.3± 2.3^{a}	113.0± 1.4^{b}	0.0± 0.0^{a}	870.6± 24.2^{a}	1.4± 0.7^{a}	857.0± 78.4^{a}	3.8± 1.0^{b}
NP	57.3± 5.6^{b}	15.5± 9.2^{a}	788.3± 37.3^{b}	1.1± 0.2^{a}	2.6± 0.7^{a}	1.2± 0.2^{a}	11.4± 4.5^{a}	54.1± 9.1^{a}	4623.5± 152.7^{b}	723.9± 23.9^{b}	266.0± 13.0^{b}	0.0± 0.0^{a}	176.0± 6.6a	1.5± 0.6^{a}	835.3± 49.2^{a}	0.1± 0.02^{a}	1311.9± 87.4^{a}	32.5± 0.6^{a}

Table S2. GC × GC-TOF-MS parameters for comprehensive profiling of fruit samples.

**Autosampler settings**	
Incubator and agitator	On
Incubator temperature	80 °C
Fill speed	50 µL/s
Injection speed	100 µL/s
Injection volume	1.5 mL
Syringe temperature	80 °C
**GC × GC-TOF-MS conditions**	
Injection mode	Splitless
Injector temperature	250 °C
Carrier gas	Helium (99.9999%)
Flow rate	1 mL/min
Primary column	Agilent DB-624UI (midpolar)
Primary column composition	6% cyanonpropyl phenyl, 94% polydimethyl siloxane
Primary column length	30 m x 250 µm x 1.4 µm
Secondary column	Restek Stabilwax (polar)

Secondary column composition	crossbond polyethylene glycol
Secondary column length	0.9 m x 250 µm x 0.50 µm
Secondary column temperature	15 °C offset primary
Modulator temperature	25 °C offset primary
Modulation	2.5 s
Hot pulse time	0.4 s
Cool time	0.85 s
Transfer line	240 °C
MS voltage	1700 V
Electron energy	70 V
Scan rate	200 spectra/s
Mass range	35-500 *m/z*
Ion source	240 °C
**Metadata pre-processing parameters**	
Baseline offset	0.5
Peak Width	15
Match Required to combine (2D)	600
Peak width (2D)	15
Minimum signal/noise (S/N)	25
Segmented Processing S/N	250
Number of hits to return	5
Minimum mol Weight	45
Maximum mol Weight	500
Mass Threshold	10
Minimum similarity match	600
**Stat Compare options**	
ChromaTof version	V4.50
Minimum similarity match	700
Maximum modulation periods apart	1
Maximum RT difference (s)	0.1
Minimum number of samples that contain the analyte	5
Minimum percent of samples in a class that contain the analyte	50

Table S3. Amino acids' single ion monitoring for mass spectrometry detector.

Amino Acid Name	Single Ion Monitoring*	Amino Acid Name	Single Ion Monitoring*	Amino Acid Name	Single Ion Monitoring*
L-Alanine	260.19	L-Histidine	326.3	L-Proline	286.2
L-Arginine	345.3	L-Isoleucine	302.3	L-Serine	276.2
L-Aspartic Acid	304.2	L-Leucine	302.3	Taurine	296.2
L-Cysteic Acid	340.3	L-Lysine	317.3	L-Threonine	290.2
L-Cystine	411.4	L-Methionine	320.3	L-Tryptophan	375.3
L-Glutamic Acid	318.2	L-Norleucine	302.3	L-Tyrosine	352.3
Glycine	246.17	L-Phenylalanine	336.3	L-Valine	288.3

*Total mass of amino acid and derivatising agent (molecular weight = 171.10)

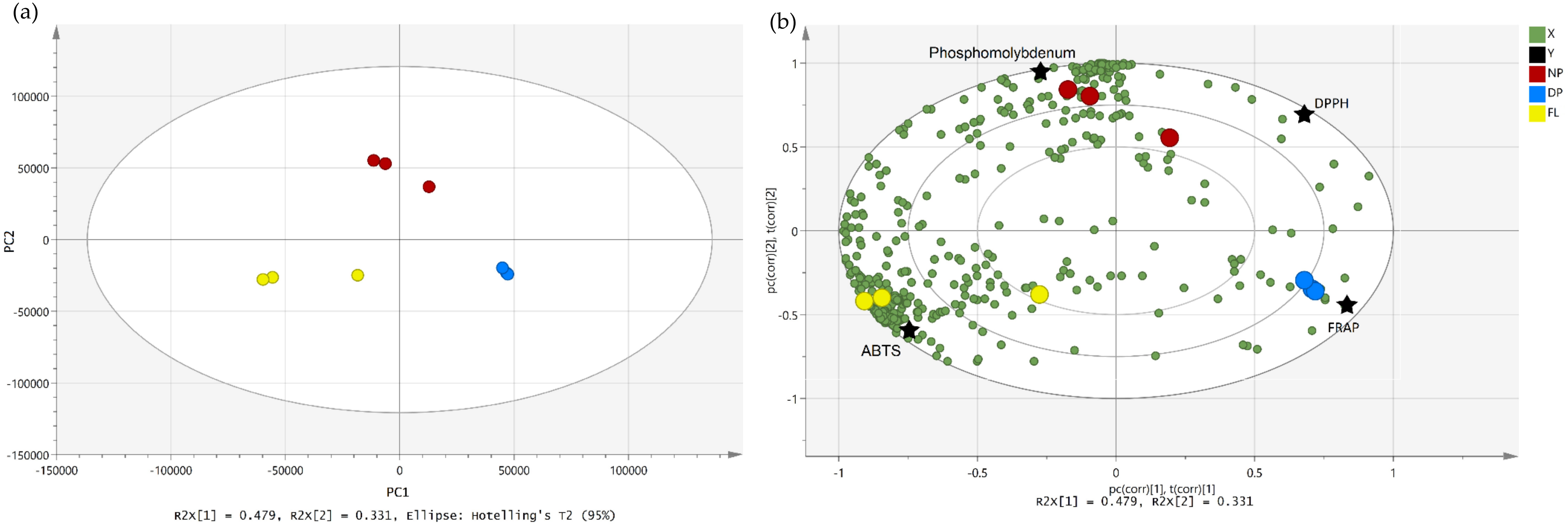

Figure S1. (a) Partial least square (PLS) score plot based on GC x GX-TOFMS data. (b) PLS biplot plots showing correlation between identified aroma compounds with antioxidant activity. X=compounds, Y=antioxidant activity.

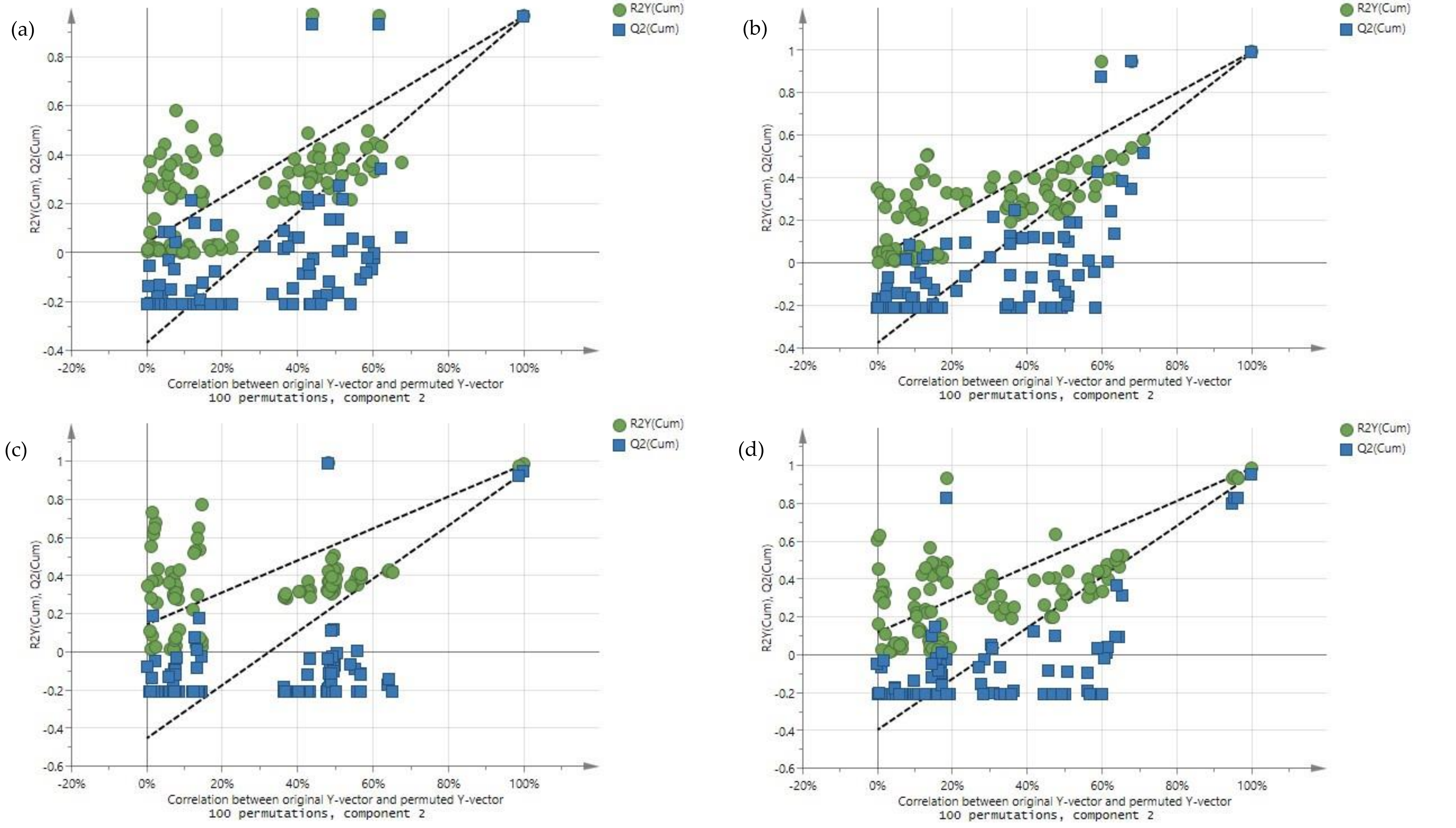

Figure S2. Permutation test of the PLS model based on UHPLC-QqQ-TOF-MS/MS data (a) ABTS; (b) DPPH; (c) FRAP; (d) Phosphomolybdenum assays

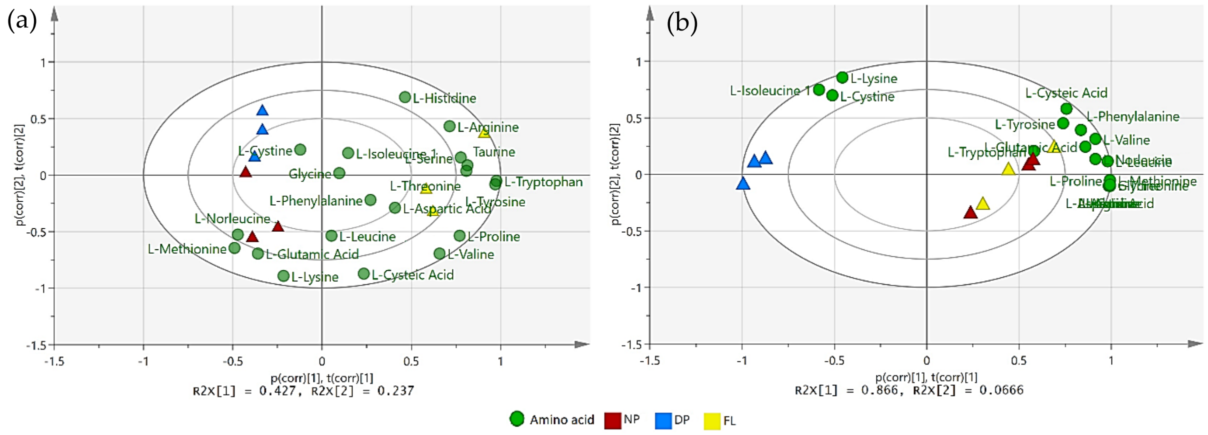

Figure S3. Biplot association of (a) free amino acid; (b) hydrolysed protein amino acid in Davidson's plum (DP), finger lime (FL) and native pepperberry (NP).

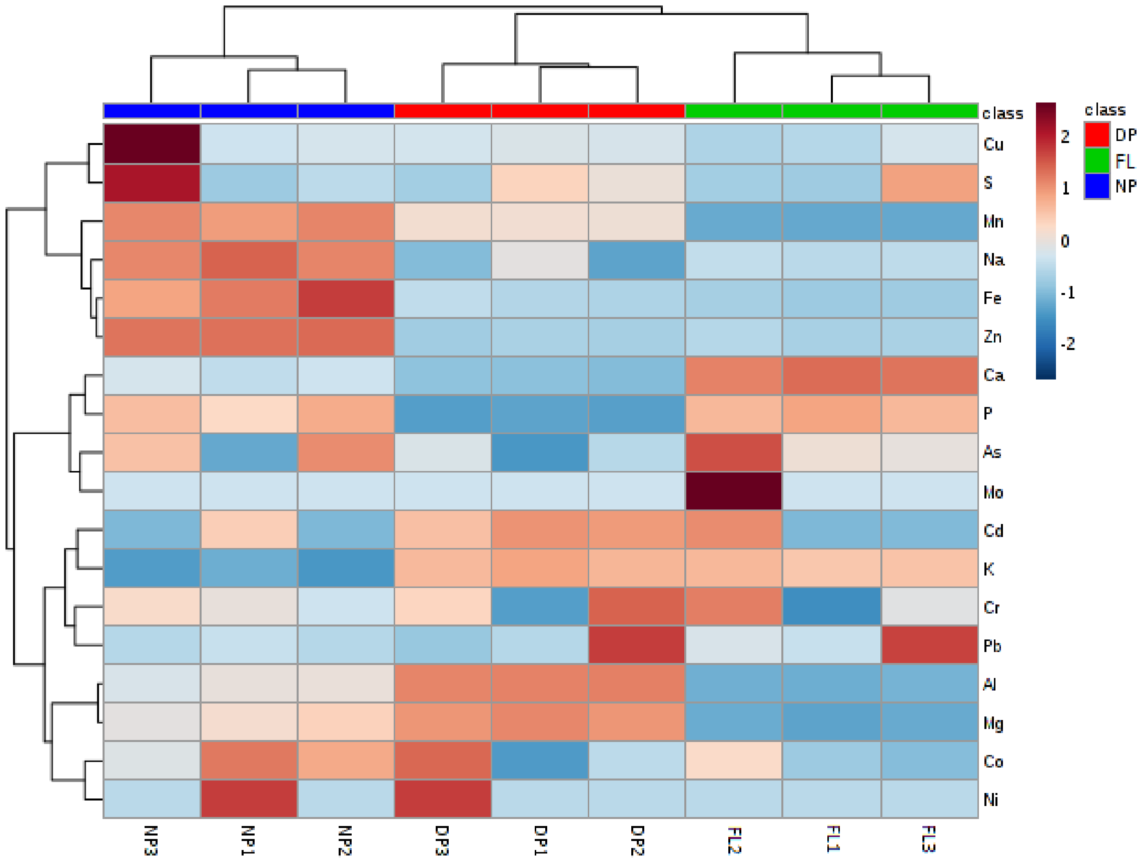

Figure S4. Heat map of 18 mineral nutrients and heavy metals found in the fruit samples.
